# A Genome Wide Association Scan of Bovine Tuberculosis Susceptibility in Holstein-Friesian Dairy Cattle

**DOI:** 10.1371/journal.pone.0030545

**Published:** 2012-02-15

**Authors:** Emma K. Finlay, Donagh P. Berry, Brian Wickham, Eamonn P. Gormley, Daniel G. Bradley

**Affiliations:** 1 Department of Genetics, Smurfit Institute, Trinity College Dublin, Dublin, Ireland; 2 Animal and Grassland Research and Innovation Centre, Teagasc, Moorepark, Fermoy, County Cork, Ireland; 3 Irish Cattle Breeding Federation, Shinagh House, Bandon, County Cork, Ireland; 4 School of Agriculture, Food Science and Veterinary Medicine, Veterinary Sciences Centre, University College Dublin, Belfield, Dublin, Ireland; Hopital Raymond Poincare - Universite Versailles St. Quentin, France

## Abstract

**Background:**

Bovine tuberculosis is a significant veterinary and financial problem in many parts of the world. Although many factors influence infection and progression of the disease, there is a host genetic component and dissection of this may enlighten on the wider biology of host response to tuberculosis. However, a binary phenotype of presence/absence of infection presents a noisy signal for genomewide association study.

**Methodology/Principal Findings:**

We calculated a composite phenotype of genetic merit for TB susceptibility based on disease incidence in daughters of elite sires used for artificial insemination in the Irish dairy herd. This robust measure was compared with 44,426 SNP genotypes in the most informative 307 subjects in a genome wide association analysis. Three SNPs in a 65 kb genomic region on BTA 22 were associated (*i.e.* p<10^−5^, peaking at position 59588069, p = 4.02×10^−6^) with tuberculosis susceptibility.

**Conclusions/Significance:**

A genomic region on BTA 22 was suggestively associated with tuberculosis susceptibility; it contains the taurine transporter gene SLC6A6, or TauT, which is known to function in the immune system but has not previously been investigated for its role in tuberculosis infection.

## Introduction

Bovine tuberculosis (TB) is a serious cattle disease, caused by infection with *Mycobacterium bovis*. It costs an estimated $3 billion annually in global agricultural losses [1] and is the fourth most important livestock disease worldwide [2]. *M. bovis* displays strong geographic localisation, most likely due to a series of clonal expansions [3]. Despite an eradication programme in operation since 1954 the annual animal incidence of bovine tuberculosis in Ireland remains approximately 0.5% [4]. More than 99% of the TB cases found in Ireland and the United Kingdom are part of a single clonal complex [5]. While most human tuberculosis is caused by the closely related pathogen *Mycobacterium tuberculosis, M. bovis* can also cause infections in humans [6]. Knowledge of resistance to the disease in cattle may provide insights into the global medical problem of human tuberculosis, as the immune response of cattle to mycobacterial infection bears a closer resemblance to that in humans than it does in mice [7].

Genetic variation in susceptibility to tuberculosis has been observed in cattle. Early and recent studies indicated higher resistance to TB among *Bos indicus* than *Bos taurus* [8], [9]. Also, certain pedigree lines of cattle show greater and lesser susceptibility to the disease [10]. Estimates of the heritability of reponse to *M. bovis* PPD (purified protein derivative) in Irish herds were up 0.276 [11] while heritability of TB susceptibility in British herds was estimated as 0.18+/−0.04 [12]. Moreover field studies are likely to underestimate heritability due to unequal exposure to the disease, incomplete test sensitivity and errors in both data recording and parentage [13]. Under the more controlled circumstances of experimental infection and slaughter to count lesions in the lungs a heritability of 0.48+/−0.096 was calculated in farmed red deer [14].

Susceptibility to tuberculosis is a complex phenotype. Differences in the management of cattle, climate and geographical region, age and reproductive status can all influence exposure to infection and probability of disease progression among individuals and herds [10]. The development of the disease is influenced by bacterial, host and environmental factors.

Both the innate and adaptive immune systems are involved in the host defence against tuberculosis and mycobacteria use a range of mechanisms to evade and inhibit destruction [15], [16]. Many studies have sought to dissect genetic influences on susceptibility, incorporating linkage studies, candidate gene association, whole genome association studies, admixture mapping, epigenetics, copy number variation, gene-gene interaction in the host and gene-strain interaction between the host and mycobacterium [17]. Genome wide searches for genes linked to TB susceptibility have been performed in mice, cattle and humans and several genes have been identified and validated in different experiments. For example cytokines and chemokines and their receptors, SLC11A1, CD209, DC-SIGN, and pattern recognition receptors including the toll-like receptors have all been implicated in the genetic response [15], [16], [18], [19], [20], [21], [22], [23]. A systems biology analysis approach to infection by *Mycobacterium tuberculosis* has also proved useful in integrating genomic studies of the pathogen and host and their interactions and metabolic pathways [24].

Control of bovine TB is dependent on testing of herds to detect chronic and subclinical infections, and the slaughter of infected animals. As *M. bovis* multiplies quite slowly and only cattle in an advanced stage of infection or challenged with high infective doses tend to show high circulating levels of antibodies against *M. bovis* [25], the predominant immune response to *M. bovis* in cattle is mounted by T lymphocytes [26].

The standard intradermal tuberculin test consists of simultaneous injections of bovine and avian purified protein derivative tuberculins into the skin and comparison of the swelling caused by an inflammatory response. Estimates of the test sensitivity range between 72% and 100% with median values of 80% and 93.5% for standard and severe interpretations and specificity of between 78.8% and 100% with a median of 99.5% [27]. Both the complexity of the phenotype and imprecision in test methods present a challenge to genome wide association studies (GWAS). In order to increase the power of the analysis we calculated a composite phenotype for genetic merit (estimated breeding value using a sire model, EBV) for TB susceptibility in sires based on disease incidence in daughters, as measured by skin test responses to bovine and avian tuberculin PPD.

EBVs are predictions of the genetic value of an individual, based on the phenotypes measured in their relatives. They may be used as a summary phenotype in GWAS (e.g. [28], [29]). Here, EBVs were calculated using a sire model, incorporating relevant environmental factors and only daughter information was used, not information from other pedigree relationships. This gave a powerful composite phenotype for each individual, which we used in a GWAS with 54001 SNP genotypes. Three consecutive SNPs on chromosome 22 give an association with TB, one within and two near the gene SLC6A6 or TauT.

## Results

### 1. Genotype quality assurance and population structure analysis

Genotyping was performed on 1004 Holstein-Friesian sires used in the Irish herd using the Illumina Bovine SNP50 BeadChip. After two rounds of quality control 44426 markers and 986 samples passed all criteria. 307 of these samples had informative EBVs for TB susceptibility and only these were used in analysis of TB susceptibility.

**Figure 1:**
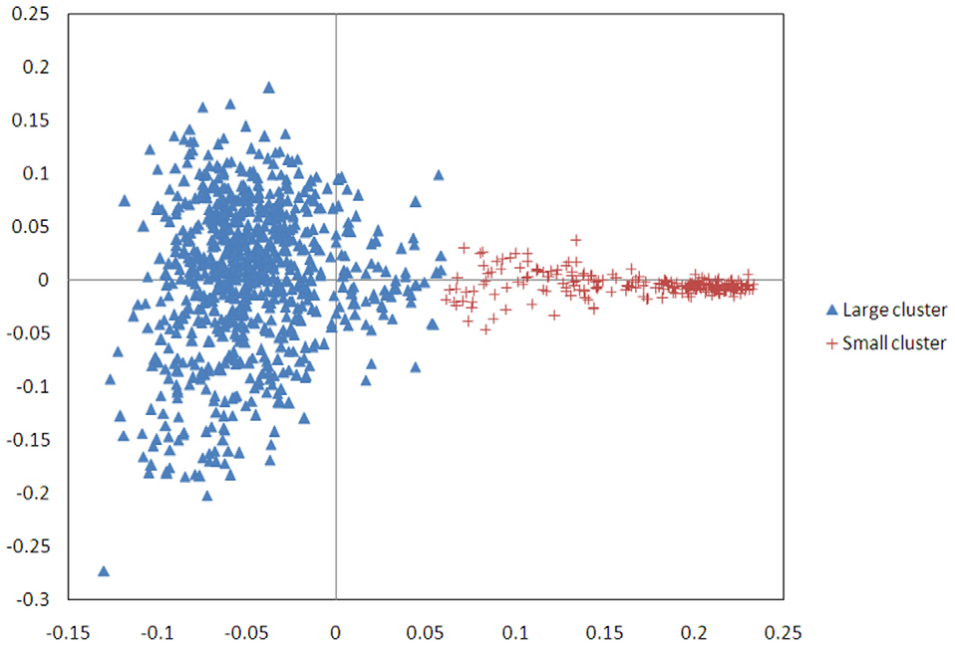
Multidimensional scaling (MDS) analysis of an Identity by State (IBS) matrix of 986 samples, divided into two clusters which reflect differing proportions of Holstein and Friesian ancestry. Both phenotypes and genotypes were adjusted for the population structure before analysis.

Multi dimensional scaling analysis of an identity by state (IBS) matrix of the samples revealed population substructure among the Holstein-Friesian samples (Figure 1). This correction uses the observed proportion of IBS alleles between each pair rather than that predicted from the pedigree relationship. When objectively divided into two clusters and the proportion of Holstein and Friesian ancestry in each sample examined, one cluster of 777 samples had averages of 97.8% Holstein, 2.2% Friesian while the small cluster averaged 23.2% Hostein, 76.8% Friesian. The 307 sires with EBVs showed a similar pattern, two clusters of 253 sires (average 97.8% Holstein, 2.2% Friesian) and 54 sires (average 24% Holstein and 76% Friesian) (Figure S1). The distribution of EBVs is not significantly different between the two clusters (Figure S2). No outliers were identified by the MDS so all EBV informative samples were included in the genome wide analysis. Both phenotypes and genotypes of the 307 samples with EBVs were adjusted for the revealed population structure.

### 2. Genome Wide Association Analysis

EBVs were calculated as in [11], incorporating data from daughters present in an episode of TB in a herd incorporating at least two infected animals and including herd/episode, month of calving of the cow, and an interaction between year of herd-test and month of herd-test as fixed effects.

**Figure 2:**
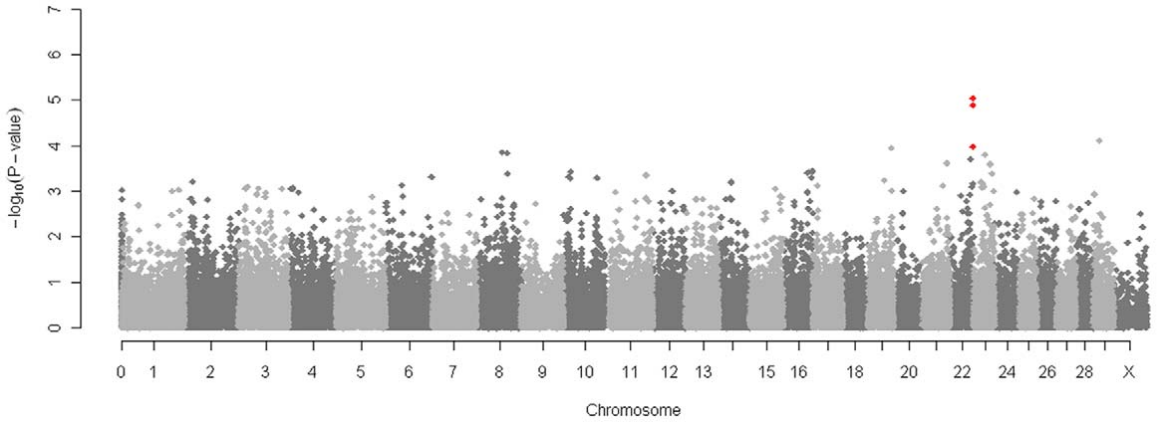
Manhattan plot displaying the results (−log 10 of p-values) of the genome wide scan with respect to genomic position. Three SNPs on chromosome 22 attain chromosome wide significance and are indicated with red dots.

**Figure 3:**
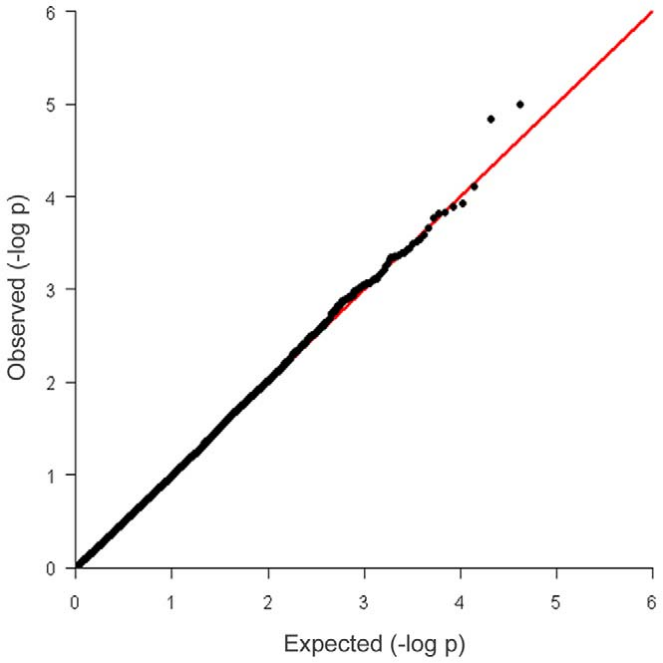
Q-Q plot of observed p-values against expected p values.

**Figure 4:**
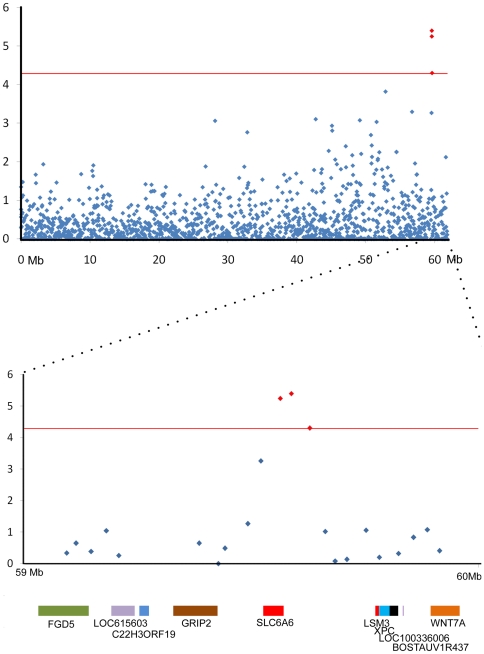
Scatter plot of the p values on chromosome 22 and a 1 Mb region surrounding the three most significant SNPs. Significance (−log 10 of p-values) is plotted against position along the chromosome and the three SNPs above the threshold of chromosome wide significance (the red line) are indicated in red. The 1 Mb region surrounding these SNPs is shown in more detail and superimposed on the genes found in the region. One of the SNPs lies within the first intron of the gene SLC6A6 and the other two are upstream (plot generated using build Btau4.2 on animalgenome.org).

Genome wide analysis of the association between the TB EBV and each SNP was performed after correcting for the underlying population structure. After principal component analysis of the IBS matrix both EBV and genotype were normalised on the axes of variation (principal components) and the correlation computed between corrected phenotype and genotype to remove the effects of similarity due to shared ancestry. After this correction the genomic inflation factor λ was 1 and the observed test statistics did not require adjustment. The genome wide Manhattan plot displaying the resulting p-values with respect to genomic position is shown (Figure 2). The distribution of p-values for each SNP was also compared to the expected distribution in a Q-Q plot where some deviation from expectation was observed at higher values(Figure 3).The three most strongly associated SNPs, all on chromosome 22, were significant at a chromosome wide level with p-values of less than the chromosome wide significance threshold 5.19×10^−5^ (Figure 4). All had high call rates of >0.99 and MAF of between 0.07 and 0.1 (Table 1).

**Table 1:** SNPs with chromosome wide (p<5.19×10^−5^) significant associations to TB EBV.

SNP	BTA	Position	p-value	Call rate	P.11	P.12	P.22
ARS-BFGL-NGS-60576	22	59588069	4.02×10-06	0.999	29	140	137
ARS-BFGL-NGS-21481	22	59563696	5.67×10-06	0.999	128	150	28
ARS-BFGL-NGS-102776	22	59628616	5.0×10-05	0.999	20	126	160

BTA: Bos taurus Chromosome.Position: base pair on the chromosome. P-value: p-values with one degree of freedom. CallRate: proportion of samples in which SNP was successfully called. P11: number of samples with AA genotype. P.12: number of samples with AB genotype. P.22: number of samples with BB genotype.

These SNPs on chromosome 22 at positions 59588069, 59563696 and 59628616 (BTAU4.0 assembly (ftp://ftp.hgsc.bcm.tmc.edu/pub/data/Btaurus) had corrected p-values of 4.02×10^−6^, 5.67×10^−6^ and 5×10^−5^ and explain 0.000005%, 0.000027% and 0.000003% of the phenotypic variance respectively. They are in linkage disequilibrium, two strongly, (ARS-BFGL-NGS-21481 and ARS-BFGL-NGS-60576, r^2^ = 0. 9184) and the third less so (ARS-BFGL-NGS-21481 and ARS-BFGL-NGS-102776, r^2^ = 0.7284, ARS-BFGL-NGS-60576 and ARS-BFGL-NGS-102776, r^2^ = 0.7932). ARS-BFGL-NGS-21481 lies within the first intron of the gene SLC6A6, *Bos taurus* solute carrier family 6 (neurotransmitter transporter, taurine) member 6 or Taurine Transporter TauT (Figure 4) and the other two SNPs lie 16.9 kb and 57.4 kb upstream of it. SNP positions are slightly different in the UMD3 assembly but these remain contiguous and SLC6A6 the closest gene. The three SNPs form a bell shaped peak with another SNP that approaches significance with a p-value of 5.48×10^−4^.

When the analysis was repeated on the two sire clusters identified in the MDS analysis of the IBS matrix two of the same SNPs were significantly associated with the phenotype in the large cluster and none in the small cluster, probably due to small sample size. The top four SNPs identified in the large (predominantly Holstein) cluster included the three chromosome 22 SNPs ARS-BFGL-NGS-60576 (p = 1.06×10^−5^) , ARS-BFGL-NGS-21481 (p = 1.11×10^−5^) and the non-significant ARS-BFGL-NGS-102776 (p = 1.22×10^−4^) and a SNP on chromosome 21, ARS-BFGL-NGS-28332 (p = 1.73×10^−5^) which lies within the predicted gene chr21.1379 and within 300 kb of BDKRB2, bradykinin receptor B2. Analysis of the smaller (predominantly Friesian ) cluster alone found no SNPs with p<1×10^−4^.

## Discussion

TB infection is a challenging phenotype for GWAS, given that an animal's probability of developing the disease depends on exposure to infection, plus many epidemiological factors, and the tests to detect infection are imperfect. However, EBVs for each sire summarise information on many daughters and the EBV calculation process involved data editing to maximise the probability that cattle used in the genetic evaluation had been exposed to the infection. Thus, the power of the study is much greater than would be a field study involving a similar number of individuals as cases and controls. The same SNPs show the strongest association in the subgroup of animals with a higher Holstein component but not in the smaller subgroup with a significant Friesian component. This may be due to breed differences or the small sample size of the Friesian subgroup.

This analysis has identified a genetic region that may have a role to play in susceptibility to bovine tuberculosis. It contains three significant SNPs and another approaching significance. These SNPs are located within a 65 kb region of the genome, form a bell shaped peak suggesting a non random association, and one lies within an intron and two less than 60 kb upstream of the gene SLC6A6, the taurine transporter TauT. The SNPs do not reach genome-wide Bonferroni-corrected significance but this may be conservative. Bonferroni correction inflatesType II errors, particularly when sample size is low, because SNPs are in linkage disequilibrium across the genome violating the assumption that all comparisons are independent. These SNP associations are significant at a chromosome wide level which is suggestive of a region implicated in tuberculosis susceptibility.

The biology of the nearest gene gives some support of its involvement. Taurine is the most abundant amino acid in mammals. Its zwitterionic nature means it cannot pass through lipid layers so the taurine transporter TauT can create concentration gradients across membranes. Taurine transporter knockout mice show severely decreased taurine levels in many body organs and systems, along with many pathological features, severe skeletal muscle impairment, hepatitis and blindness [30]. Knockout mice also show important immunological differences to wild type mice, for example they are more sensitive to ultraviolet B induced immunosuppression [31].Also, blood-stage malaria from which wild type mice fully recover is fatal in the knockout mice [32]. Here lower levels of taurine in the cells and blood critically affected the stability of cells and resulted in increased parasite loads and inflammatory responses. Tumor necrosis factor α (TNFα), a prominent inflammatory mediator in the innate immune system has been shown to increase taurine uptake and TauT mRNA expression in intestinal cells [33]. When monocyte derived macrophages are experimentally infected with *Mycobacterium bovis*, TauT expression shows a significant downregulation (Professor David MacHugh, personal communication).

Taurine deficiency has been linked to immune system abnormality and its presence to prevention of inflammation in animal models [34]. Taurine maintains phagocytic and microbicidal function in aged neutrophils [35]. It is an anti-inflammatory factor in intestinal epithelial cells and it is thought that increasing the taurine concentration in response to TNFα may counteract the inflammatory response caused by pro-inflammatory cytokines produced in response to TNFα.

Taurine also has an important function in host response to bacterial pathogens. Hypochlorous acid (HOCl) is involved in bacterial killing but can also damage host cells. Taurine reacts with HOCl to form taurinechloramine (TauCl), a long lived stable oxidant, which maintains bactericidal activity but is less toxic to host tissue than the indiscriminate HOCl . TauCl has a role in regulation of proinflammatory mediator release by the macrophage as well as a bactericidal action, modulates TNF α production and inhibits the production of nitric oxide, prostaglandin E_2_, interferons and TNF-α and the proliferation of lymphocytes [36], [37], [38], [39], [40]. Of several chloramines examined TauCl was unique in its combination of structural stability and inhibition of proinflammatory cytokine production and the least toxic to cells [41].

In sum, the suggestive genomic localisation of TB susceptibility to the gene TauT by GWAS is supported by several strands of evidence suggesting significant immunological roles for TauT and its substrate taurine.

## Materials and Methods

### Genotype data

Genomic DNA was isolated from semen obtained from the National Cattle Breeding Center, Enfield, Co. Meath, Ireland of Holstein-Friesian bulls used for artificial insemination. The semen was washed twice in phosphate buffered saline (pH 7.4), cell pellets were harvested by centrifugation and re-suspended in 450 µL of pre-warmed extraction buffer (10 mM Tris pH 8, 10 mM EDTA pH 8.0, 1% SDS, 100 mM NaCl) and 15 µL of 2-Mercaptoethanol was then added. Samples were incubated at 55°C for 15 minutes followed by the addition of 10 µL Proteinase K (20 mg/ml). Lysis occurred following an overnight incubation at 60°C. DNA was extracted using the Maxwell® instrument (Promega Corp., Madison WI) and according to the manufacturer's instructions.

All animals were genotyped commercially using the Illumina BovineSNP50 BeadChip [42], containing 54001 SNPs with an average spacing of 51.5 kb and a median spacing of 37.3 kb based on the BTAU4.0 assembly (ftp://ftp.hgsc.bcm.tmc.edu/pub/data/Btaurus).

### Phenotypic data

Estimated breeding values (EBV), which are estimates of genetic merit for an animal for a given phenotype were calculated, using a sire model, in ASREML [43] from 14,013 Irish Holstein-Friesian cows with single intradermal comparative tuberculin test results. Relationships among sires were ignored so that the only contribution to a sire's genetic merit was its daughters' phenotypes, thereby avoiding double counting. The data, including editing criteria imposed, are described in detail by Bermingham et al. [44]. In brief, data from the years 2000 to 2005 were used and only cows present in a herd during a period of infection where at least 2 cows showing evidence of infection (standard interpretation of the single intradermal comparative tuberculin test) were retained; this ensured a high risk of exposure to *M. bovis* in the herd. Animals that moved into the herd during, or within 6 weeks of the start of a period of infection were removed to maximize the likelihood of equalized, within herd, *M. bovis* exposure, as it takes 3 to 6 weeks post infection for cattle to develop a positive reaction to the SICTT [27].

Fixed effects included in the sire model for the estimation of breeding values were as described by in [11] and consisted of herd - episode, month of calving of the cow, and an interaction between year of herd-test and month of herd-test. The pedigree of each sire was traced back at least four generations and included as a random effect in the model.

The 307 sires used had EBVs calculated using data from between 1 and 1046 daughters, with a mean number of 34,median of 9 and mode of 4 daughters.

### Quality control

Genotype quality control was performed using the “check.marker” function of the R statistical environment package GenABEL [45]. SNPs with more than 5% missing data, a minor allele frequency of less than 0. 27% or significantly out of Hardy Weinberg Equilibrium (HWE) (FDR = 0.2) or with a sex chromosome genotype incompatible with the sample's sex were discarded, as were samples with more than 5% missing data or more than 95% identity by state (IBS) with another sample.

In the first round 1567 markers were excluded because of extremely call rate <0. 95 , 5840 markers were excluded as having low (<0.27%) minor allele frequency. 121 X-linked markers were likely to be autosomal (odds >1000), 1455 X/Y/mtDNA impossible heterozygotes and female Ys were set as missing. One sample was excluded because of a low (<95%) call rate and one was excluded because of IBS> = 0.95. 75 male were likely to be female (odds >1000). In the second round 13 markers were excluded due to a low call rate, 5302 markers were excluded because they were out of HWE. 1911 heterozygous X-linked male genotypes were found, 756 X/Y/mtDNA impossible heterozygotes and female Ys were set as missing and 26 male were likely to be female (odds >1000). Two samples were excluded due to low call rates(<95%). Due to the way quality control is performed sequentially the sum of all categories excluded is greater than the number of SNPs or samples excluded, some were excluded for multiple reasons.

Following editing 44426 SNPs from 986 animals with both genotype and phenotypic data remained for inclusion in the association analysis.

### Association analysis

Multi dimensional scaling (MDS) analysis of an identity by state (IBS) matrix of the samples was used to reveal population substructure. Samples were objectively divided into two clusters using the k-means method [46] and the proportions of Holstein and Friesian for the samples in each cluster quantified using theHolstein and Friesian component in the Holstein-Friesian breed society database and is based on pedigree information stored (at least three generations on each animal)

The genomic kinship matrix was used to derive axes of genetic variation (principal components) and then both the EBV and genotypes were adjusted onto these axes [47] using the egscore command of GenABEL [45]. The association between the breeding value and each SNP was analysed using a linear model with the SNP genotype and the axes of variation as predictors. The corrected genotypes were defined as the residuals from regression of the genotypes on the orthogonal axes of variation before correlation between phenotype and genotype was corrected.

A genome wide Bonferroni correction may result in high false negatives because it ignores correlations between markers and leads to an overly conservative correction, a problem which intensifies as the marker density increases [48]. Thus instead of a genome wide Bonferroni correction of p<1.21×10^−6^ (0.05/the total number of SNPs) associations were tested at the chromosome-wide significance level (p<0.05/the number of SNPs on the chromosome) as in [49]. The 5% chromosome wide significance threshold ranged from 1.91×10^−5^ on chromosome one to 6.58×10^−5^ on chromosome twenty eight.

The proportion of variance explained by SNPs was estimated using the formula V = 2pqa^2^ where p and q are the frequencies of the major and minor alleles and a is the allelic substitution effect [50].

## Supporting Information

Figure S1 **The distribution of samples with EBVs within the Multidimensional Scaling (MDS) plot created from an Identical by State matrix of all samples.** If only the samples with EBVs are included in the MDS analysis and then the samples are objectively divided into two clusters one contains 253 sires (average 97.8% Holstein, 2.2% Friesian) and 54 sires (average 24% Holstein and 76% Friesian).
(DOCX)

Figure S2 **EBV distributions in all samples and in the two clusters identified by dividing an MDS analysis of an Identical by State matrix.** None of the pairwise comparisons of distributions were significantly different (Welch Two Sample t-test. All samples v.s Cluster one, t = −0.24 p = 0.81. All samples v.s Cluster two, t = −0.5 p = 0.61. Cluster one v.s Cluster two t = −0.31 p = 0.76).
(DOCX)
