# Supplementary material for: A Genome Wide Association Scan of Bovine Tuberculosis Susceptibility in Holstein-Friesian Dairy Cattle
Source: PLoS One. 2012 Feb 15;7(2):e30545. doi: 10.1371/journal.pone.0030545 (PMC3280253; doi:10.1371/journal.pone.0030545)
Supplement: Figure S1 — The distribution of samples with EBVs within the Multidimensional Scaling (MDS) plot created from an Identical by State matrix of all samples. If only the samples with EBVs are included in the MDS analysis and then the samples are objectively divided into two clusters one contains 253 sires (average 97.8% Holstein, 2.2% Friesian) and 54 sires (average 24% Holstein and 76% Friesian). (DOCX) [file pone.0030545.s001.docx]

The distribution of samples with EBVs within the Multidimensional Scaling (MDS) plot created from an Identical by State matrix of all samples.If only the samples with EBVs are included in the MDS analysis and then the samples are objectively divided into two clusters one contains 253 sires (average 97.8% Holstein, 2.2% Friesian) and 54 sires (average 24% Holstein and 76% Friesian).
