# Supplementary material for: A Genome Wide Association Scan of Bovine Tuberculosis Susceptibility in Holstein-Friesian Dairy Cattle
Source: PLoS One. 2012 Feb 15;7(2):e30545. doi: 10.1371/journal.pone.0030545 (PMC3280253; doi:10.1371/journal.pone.0030545)
Supplement: Figure S2 — EBV distributions in all samples and in the two clusters identified by dividing an MDS analysis of an Identical by State matrix. None of the pairwise comparisons of distributions were significantly different (Welch Two Sample t-test. All samples v.s Cluster one, t = −0.24 p = 0.81. All samples v.s Cluster two, t = −0.5 p = 0.61. Cluster one v.s Cluster two t = −0.31 p = 0.76). (DOCX) [file pone.0030545.s002.docx]

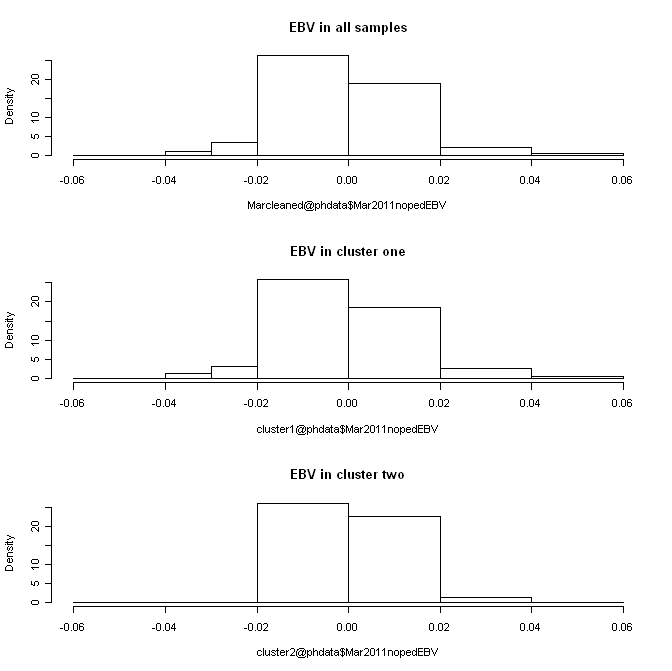


EBV distributions in all samples and in the two clusters identified by dividing an MDS analysis of an Identical by State matrix. None of the pairwise comparisons of distributions were significantly different (Welch Two Sample t-test. All samples v.s Cluster one, t =-0.24 p =0.81. All samples v.s Cluster two, t=-0.5 p=0.61. Cluster one v.s Cluster two t=-0.31 p=0.76).
